# Monthly Summary

**Published:** 1890-05

**Authors:** 


### Monthly Summary.

MOUTH-BREATHING AND THE TEETH.—Dr. Scanes Spicer read a paper at the last meeting of the Odontological Society of London, upon "Nasal Obstruction and Mouth-Breathing as Factors in the Etiology of Disorders of the Teeth." In the course of his remarks he said he had been struck with the frequency with which carious teeth were associated with obstruction of the pharynx and enlarged tonsils; so much so that he made it a routine practice to examine the teeth in all cases of nasal obstruction, and he believed that there existed a relation between them; and he further is of opinion that there is a generic relation between some cases of vaulted arch, narrow jaws, and irregular teeth, and nasal obstruction. Normally we should breathe through the nose, so as to warm and filter the air respired. All animals, savage races, and young infants do so; but a large number of adults of civilized nations breathe through the mouth, because they have some obstruction of the nasal passages—erectile tumors, permanent catarrhal affections, polypi, post-nasal adenoid growths, etc. Mouth-breathing, he said, as a predisposing cause of caries of the teeth, came into action in various ways. The teeth were exposed to a current of air of a much lower temperature than that of the body, which would tend to cause inflammation of the periosteum and pulp of a tooth; the cold dry air produced congestion of the mucous membrane, with a secretion of stringy acid mucus; and the rapid evaporation of water which takes place when the mouth is constantly open inspissated this mucus, which so formed a fertile soil for the development of micro-organisms. Again, when sleeping with the mouth open, the tongue falls back and the parotid secretion finds its way directly through the pharynx instead of bathing and washing the teeth. With reference to the so-called V-shaped maxilla, Dr. Spicer thought that many cases might be traced to mouth-breathing, the muscles of the cheek pressing unduly upon the soft alveoli when the mouth is open.—*Lancet*, Jan. 8, 1890.

---

A CASE IN PRACTICE.—*By Chas. Richardson, Fayetteville, Ark.*—Mrs. W., brought her son, aged 16, to me for examination for diseased teeth, having been referred to me by her physician. The young man was of robust health, but the left side of his face was noticably enlarged and the chin consequently thrown much to the right of the median line. I found that the inferior maxilla on the left side was considerably enlarged, externally, beginning just below the sigmoid notch and extending to a point about midway between the angle and mental process, the angle being about ½ of an inch lower and much more prominent on the left side than on the right. The soft tissues appeared as if swollen to eye and touch; but there was no inflammation and inquiry elicited the fact that there never had been a particle of soreness or uneasiness of any kind although the enlargement had been gradually increasing for a number of years. Both mother and son are highly intelligent and appreciated the importance of correct replies. As the physician thought the seat of trouble might be in the teeth, I made a careful examination with the following results: Caries in grinding surface of first and second, in molars both sides, and in median surface of left superior lateral. The teeth occlude properly except the superior centrals which close inside the lowers. They were badly decayed and were curved inwardly, which fact probably accounts for the faulty occlusion. The teeth were, with the exception of the carious ones, healthy and clean and no soreness or unpleasant effects were produced by tapping. The enlargement, as far as could be judged, had no connection with the teeth. The faulty teeth were filled except the superior centrals which were cut off and crowns fitted, which restored the articulation and, to some extent, the expression of the face. I directed the patient to await results, believing it best not to irritate the enlargement by treatment.

I would be glad to hear from others of the profession on this point, and will cheerfully answer any questions I am able in regard to it.—*Western Dental Journal.*

